# Acceptability of telehealth‐delivered Alberta Infant Motor Scale for Australian parents: A mixed‐methods analysis

**DOI:** 10.1111/dmcn.70290

**Published:** 2026-05-07

**Authors:** Kate L Rawnsley, Alicia J Spittle, Jeanie L Y Cheong, Louisa Remedios

**Affiliations:** ^1^ Department of Physiotherapy University of Melbourne Melbourne VIC Australia; ^2^ Clinical Sciences Murdoch Children's Research Institute Melbourne VIC Australia; ^3^ Newborn Research Royal Women's Hospital Melbourne VIC Australia; ^4^ Department of Obstetrics, Gynaecology and Newborn Health University of Melbourne Melbourne VIC Australia; ^5^ Department of Paediatrics University of Melbourne Melbourne VIC Australia; ^6^ Institute of Health and Wellbeing Federation University Churchill VIC Australia

## Abstract

**Aim:**

To describe the factors shaping Australian parents' acceptability of synchronous telehealth delivery of the Alberta Infant Motor Scale (AIMS), an infant gross motor assessment.

**Method:**

A mixed‐methods approach was used to describe the experiences of parents whose infants received telehealth and face‐to‐face AIMS assessments. A quantitative component used questionnaire responses (*n* = 102) and a qualitative component involved semi‐structured interviews (*n* = 13). Descriptive statistics, deductive content analysis (questionnaire data), and inductive content analysis (interview data) were used for analysis.

**Results:**

Most parents (89%) reported telehealth was an acceptable way to complete the AIMS assessment. Most (97%) would accept a telehealth appointment if a face‐to‐face assessment was not available. Four categories were identified from interview data that influenced parents' perceptions of telehealth: (1) visibility of the child; (2) accuracy of representation; (3) trust in the unfamiliar; and (4) the parental experience.

**Interpretation:**

Telehealth delivery of the AIMS is acceptable to Australian parents. However, around half of participants preferred face‐to‐face assessment. Findings suggest families be provided with choice, with clinicians supporting parents to understand the benefits and challenges of each modality, considering families' circumstances, preferences, capabilities, and access to technology.

## Graphical abstract

A mixed methods approach was used to describe the experiences of parents whose infants received telehealth and face‐to‐face Alberta Infant Motor Scale (AIMS) assessments. Although telehealth delivery of the AIMS was reportedly acceptable to Australian parents, around half of participants preferred face‐to‐face assessment. Clinical recommendations, based on parent feedback, have been provided to optimize parents' comfort and confidence using telehealth.

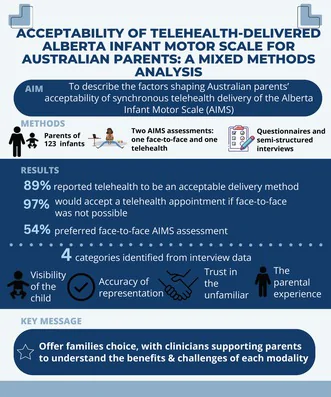

**This original article is commented by Holland on pages** 1499–1500 **of this issue.**

**Abbreviation**
AIMS: Alberta Infant Motor Scale

**What this paper adds**

- Synchronous telehealth delivery of the Alberta Infant Motor Scale (AIMS) is an acceptable method for Australian parents.
- Families can be provided with a choice between face‐to‐face and telehealth AIMS.
- Living regionally was not the primary influence for parents' acceptance of telehealth.
- Parents valued the convenience and accessibility of telehealth.

The Alberta Infant Motor Scale (AIMS) is a gross motor assessment for infants from birth until independent walking.^1^ The scale has excellent psychometric properties when used face‐to‐face,^1^, ^2^, ^3^ and agreement between face‐to‐face and telehealth delivery is high.^4^ Telehealth has the potential to improve access to developmental services for families who are unable to regularly access face‐to‐face services, owing to geographical or social circumstances.^5^, ^6^ For telehealth assessment to be implemented and sustained clinically, it needs to be acceptable to parents. Parental acceptance and satisfaction with telehealth for young children in general is high^7^, ^8^, ^9^ which is an important finding, given that parents are required to be actively involved to facilitate the consultation in this age group. Because of the variability in assessment and intervention that may occur by telehealth, it is necessary to determine acceptability specific to the context, in this case referring to the AIMS assessment.

Parental acceptability for telehealth delivery of the AIMS has previously reported to be high; however, one study,^10^ based in the Netherlands, involved pre‐recorded home videos (i.e. asynchronous telehealth). Clinical barriers to pre‐recorded videos exist such as upload requirements and reimbursement of clinicians' time to review the videos; therefore clinical implementation of this method may be limited.^11^ A more recent study used synchronous (i.e. real time) telehealth to deliver the AIMS assessment;^4^ however, it took place in the cultural context of Brazil, a middle‐income country, and used the Portuguese version of the AIMS. Owing to differences in language, sociocultural influences, environmental factors, and caregiving practices,^12^ the findings of the Brazilian study^4^ may not be generalizable to other countries. Parents' perspectives of synchronous telehealth delivery of the AIMS have not previously been explored in Australia. Thus, this study aimed to describe the factors that shape acceptability of synchronous telehealth delivery of the AIMS assessment for Australian parents.

## METHOD

### Study design

A pragmatic ontological and epistemological position^13^ led to the selection of a mixed‐methods approach to describe the experiences of parents who had received both telehealth and face‐to‐face in‐home AIMS assessments for their infants. A survey was used to capture parents' perceptions of efficacy of telehealth, while semi‐structured interviews were used to gain a deeper understanding of parental responses to both assessment experiences. The questionnaires focused on perceived acceptability, facilitators, and barriers to telehealth whereas the interview questions explored the experiences of parents during the telehealth and face‐to‐face sessions, probing further the factors that shaped acceptability. This study was reported according to the standards for reporting qualitative research.^14^

### Participants

Participants were parents of infants from metropolitan Melbourne and the regional town of Bendigo in Victoria, Australia, who had participated in a study determining the agreement between telehealth and face‐to‐face delivery of the AIMS. Infants received two AIMS assessments within 2 weeks: one face‐to‐face in their home and the other by telehealth at 4 months, 8 months, or 12 months of age. Assessment order was randomized and different clinicians conducted each assessment to limit risk of bias. Further details have been published elsewhere.^15^ On completion of both assessments, all families (117 parents of 123 infants) received a brief electronic questionnaire asking about their experiences in the study.

In addition, semi‐structured interviews were conducted with 13 of 111 parents who indicated their willingness to be interviewed. A purposive maximum variation sampling strategy^16^ was used to achieve adequate diversity and a broad representation of parental experiences on the basis of child's age, development (based on AIMS centiles), and location (metro vs regional). Ethics approval was obtained from the Human Research Ethics Committee at the Royal Women's Hospital, Melbourne, VIC, Australia (HREC/87540/RWH 22–26). Parents provided electronic informed consent to participate.

### Questionnaires

Online questionnaires (Appendix S1) were sent by e‐mail to families through the Research Electronic Data Capture (REDcap), with three reminders within a month if not completed. The questionnaires consisted of 8 to 12 questions, both open and closed in nature and took approximately 5 minutes to complete.

### Semi‐structured interviews

Interviews were arranged at a time suitable for the participant and the researcher within 2 months or less of the AIMS assessments. Interviews were limited to a suggested duration of 30 to 60 minutes, with a willingness to listen for as long as participants wished. A semi‐structured interview guide (Appendix S2) was developed and pilot tested. Interviews took place using video conferencing software (Zoom, Zoom Communications Inc., USA) or by telephone (audio). With consent, all interviews were recorded. The recording was transcribed and analysed. Reflexive journal entries were made after each interview. Participants were provided with a visual summary of the authors' analysis, allowing them to provide feedback if they wished. No repeat interviews were required.

### Analysis

Quantitative data from the questionnaires were analysed using descriptive statistics (frequencies and percentages) with STATA version 18 (StataCorp, College Station, TX, USA). Text responses to open‐ended questions were analysed using deductive content analysis with an unconstrained matrix.^17^

Content analysis using a predominantly inductive approach was used to analyse interview data following the methodology described by Elo and Kyngäs.^17^ This approach was chosen as the intention was to gather practical responses to shape future clinical practice rather than build theory. After a period of familiarization, transcripts were open coded by KLR and a list of categories was generated.^18^ Similar topics were combined under top‐order headings and an abstraction process was used to further group and refine categories. NVivo software, version 15 (Lumivero, USA) was used to assist coding and categorization. KLR is a paediatric physiotherapist and the parent of a child with additional needs, living regionally, and accessing multiple medical specialists and allied health professionals. KLR is motivated to improve the inequity of access to developmental services for regional and remote families. She acknowledges a bias towards the benefits of telehealth and believes it is one strategy to help minimize this inequity. She remained aware of this bias throughout and was open to different experiences and paid particular attention to negative cases. A selection of transcripts was open coded by a second researcher (LR) to improve trustworthiness.

## RESULTS

### Questionnaires

Between June 2023 and April 2024, 102 responses from a potential 117 parents were received (87% response rate). There were five sets of twins and one pair of siblings, thus 123 infants from 117 households received assessments. There were two parents who returned individual questionnaires for each child in a twin set, so responses were received from 100 parents; there were 17 non‐responders (85% of parents responded). Characteristics of the participants and non‐responders are outlined in Table 1. Responses were grouped into three main categories: telehealth acceptability, benefits of telehealth, and challenges of telehealth.

**TABLE 1:** Characteristics of questionnaire participants and non‐responders.

	Response received, *n*	Non‐responders, *n*
Location	Metropolitan, 69; regional, 33	Metropolitan, 9; regional, 8
Parent of multiples	6^a^	1
Infant age group	4 months, 40; 8 months, 44; 12 months, 18	4 months, 7; 8 months, 6; 12 months, 4
Caregiver level of education	Year 12,^b^ 2; post‐secondary education/training, 100	Year 12,^b^ 1; post‐secondary education/training, 15; not stated, 1
AIMS score below referral threshold^c^	Yes, 9; no, 93	Yes, 2; no, 15

Abbreviation: AIMS, Alberta Infant Motor Scale.

^a^ Six questionnaires returned from four families of multiples.

^b^ Year 12 is the final year of schooling in Australia.

^c^ Defined as a score below referral threshold on face‐to‐face AIMS assessment, namely <10th centile for 4‐month‐old group and <5th centile for 8‐month‐old and 12‐month‐old groups.

#### Telehealth acceptability

From the 102 questionnaire responses, 89% of parents agreed or strongly agreed that telehealth was an acceptable way to complete the AIMS assessment and 76% felt confident that the clinician could get the information they needed by telehealth (Table 2). Acceptability based on the participants' characteristics is shown in Table 3, with metropolitan parents responding slightly more favourably to telehealth than regional parents (94% vs 79% respectively agreed or strongly agreed that telehealth was acceptable). Most parents (97%) reported they would participate in a telehealth assessment if an in‐person appointment was not possible. Just over half of responding parents (*n* = 101; one did not respond) preferred face‐to‐face assessment (54%) while 14% preferred telehealth and 32% had no preference. Parents of infants who scored below the referral threshold on the face‐to‐face AIMS marginally favoured face‐to‐face assessment (6 out of 9; 67%) while one preferred telehealth (11%) and two had no preference (22%).

**TABLE 2:** Parents' opinions of AIMS delivery by telehealth.

Questionnaire question	Strongly agree, *n* (%)	Agree, *n* (%)	Neutral, *n* (%)	Disagree, *n* (%)	Strongly disagree, *n* (%)
In your opinion, was telehealth an acceptable way to complete your child's assessment?	22 (21.6)	69 (67.6)	7 (6.9)	3^a^ (2.9)	1^a^ (1)
Did you feel confident that the physiotherapist was getting all the information they needed to complete the assessment via telehealth?^b^	20 (19.8)	57 (56.4)	18 (17.8)	5 (5)	1 (1)
Did you feel comfortable handling your child during the telehealth assessment?	53 (52)	44 (43.1)	4 (3.9)	1^c^ (1)	0 (0)

Abbreviation: AIMS, Alberta Infant Motor Scale.

^a^ Parents reported this was due to difficulty following instructions (*n* = 3), infant not cooperating (*n* = 1), infant distracted by screen (*n* = 2), and/or difficulty managing twins without extra hands (*n* = 1).

^b^ One response missing from the data for this question, (*n* = 101).

^c^ Parent stated it was hard to move the infant and hold the camera at the same time.

**TABLE 3:** Is telehealth an acceptable way to complete the AIMS assessment?

Subgroup	Strongly agree, *n* (%)	Agree, *n* (%)	Neutral, *n* (%)	Disagree, *n* (%)	Strongly disagree, *n* (%)
Metropolitan parents	16 (23.2)	49 (71.0)	2 (2.9)	1 (1.5)	1 (1.5)
Regional parents	6 (18.2)	20 (60.6)	5 (15.2)	2 (6.1)	0 (0)
Parents of 4‐month‐olds	7 (17.5)	29 (72.5)	3 (7.5)	1 (2.5)	0 (0)
Parents of 8‐month‐olds	13 (29.6)	27 (61.4)	3 (6.8)	0 (0)	1 (2.3)
Parents of 12‐month‐olds	2 (11.1)	13 (72.2)	1 (5.6)	2 (11.1)	0 (0)

*Note*: Values given are responses from parents on the basis of characteristics.

Abbreviation: AIMS, Alberta Infant Motor Scale.

#### Benefits of telehealth

Open‐ended questions asked parents to share the perceived benefits of completing the AIMS assessment by telehealth. Responses are summarized in Table 4 with supporting de‐identified quotes.

**TABLE 4:** Benefits of telehealth AIMS assessment.

Benefits	Number of times mentioned	Supporting quotes (open‐ended survey responses)
Convenience	71	‘Convenient, no travel time, can work around child's naps easier, didn't require much effort on my behalf. It was quick.’ Q007
Child more comfortable in own environment	33	‘My child appeared more confident and comfortable as she wasn't overwhelmed having a new person in her space. I felt that her movement during the assessment was a better reflection of her and her abilities compared to the in‐person.’ Q125
Improves access, for example rural, immunocompromised	22	‘Being able to access specialist practitioners in regional areas without needing to wait as long.’ Q032
Do not have to cancel when unwell	5	‘if baby/parent unwell or worried about exposing child to illness, telehealth avoids this.’ Q049
Parents more involved in assessment	4	‘I also liked that I was more hands‐on during the telehealth session, I felt that I will remember more information as I was more of an active member of the session.’ Q031
Less cost to parents, for example transport/parking	3	‘Minimisation of cost (e.g. time taken off work, time to travel, parking costs, fuel costs).’Q067
Benefits to clinician, for example travel time for home visit	3	‘… [telehealth] means that the physios can also see more patients in one day, thus helping more people!’ Q014
More flexible for scheduling	2	‘My infant was at the very end of her wake window and was clearly tired. The physiotherapist was able to offer some further time later in the day after the baby had had a nap to finish off the assessment. This kind of flexibility would be unique to telehealth therapy.’ Q085
Other, for example ease of phone/laptop; move screen to follow child, prior information helpful	3	‘Emailed info helped set it up for success.’ Q069

Abbreviation: AIMS, Alberta Infant Motor Scale.

#### Challenges of telehealth

An open‐ended question asked parents, ‘in your opinion, what were the challenges (if any) of a telehealth AIMS assessment?’. Parents described challenges that they either experienced or saw the potential for occurring in the future. Responses are summarized in Table 5, with a de‐identified quote supporting each challenge. While technology issues were commonly reported as a challenge, in several cases this was hypothesized as a potential issue rather than the participant's own experience. Technology issues experienced in the study were minor and did not prevent assessments from occurring, such as a lag in audio transmission, brief interruptions in connection, or a delay logging into the video conferencing platform.

**TABLE 5:** Hypothesized or actual challenges of the telehealth AIMS assessment.

Challenges	Number of times mentioned	Supporting quotes (open‐ended survey responses)
Capturing child with the camera	36	‘Capturing all of baby's movements and whole body onto a screen (especially feet to see if they are taking a step).’ Q045
Technology or internet connection issues	23	‘At times there was a pause between the conversation being received, so that was tricky.’ Q08
Facilitating the assessment	20	‘I was nervous I wasn't moving [my child] or the toys to get him to perform the skills they were looking for but the assessor was reassuring throughout which helped.’ Q021
Child distracted by the screen	17	‘My baby was very distracted by the computer screen and thus wasn't participating as well as she was capable of.’ Q062
Rapport building	11	‘I didn't ask any questions because I felt more comfortable in the [face‐to‐face].’ Q076
No challenges experienced	11	‘I had a wonderful experience and we didn't experience any challenges.’ Q061
Concerns therapist could not see everything	5	‘Hard to tell if the assessor could see the detail required to make an assessment on telehealth.’ Q123
Having a suitable space and/or toys	5	‘Finding the right space at home for the appointment (we have a small apartment, so finding a place where we could position the laptop and give the physio the right visibility was a bit of a challenge, but this is minor).’ Q001
Dependent on skills of clinician and parent	3	‘It would be dependent on the strength of the assessors and the parents communication skills ie describing what the parent needs to do.’ Q028
Other, for example hypothesized language or education barriers	3	‘There may be challenges with language or level of education and a patients capacity to understand and action the direction given by the clinician.’ Q078
Parents not knowing what to expect	2	‘When doing telehealth it might be good to have a rough diagram showing the best angle to set up the laptop, and the proximity to the play area and toys, for people who are more visual learners. I think the inclusion of the prop/baby doll for the physio/assessor to demonstrate on was great for the same reason.’ Q010
Lacks novelty factor for child (i.e. no new face or new toys)	2	‘Because baby was more tired and was only with me (mum), it was difficult to engage them towards the end as they were getting fussy. Where if physio was present, a new person may have been enough of a distraction to keep baby engaged in play/testing.’ Q009
Cannot see AIMS assessment scoresheet	2	‘Given the parent is acting as a semi‐assessor it would have been good to know what things are actually on the AIMS, so as a parent you probably know what may evoke certain movements or positions from the baby.’ Q123

Abbreviation: AIMS, Alberta Infant Motor Scale.

### Semi‐structured interviews

Semi‐structured interviews were conducted between October 2023 and April 2024. Eighteen parents were offered an interview, of whom 13 participated (two were unavailable and three did not respond to the invitation). Interviews were of 30 to 50 minutes' duration except for one, which was 15 minutes owing to the participant's availability. Code saturation^19^ was reached with the 13 completed interviews. Interview participants were selected to achieve diversity in parents' characteristics, geographical location, child's age, level of developmental concern, and views on telehealth (on the basis of survey responses). The characteristics of participants interviewed are outlined in Table 6. Interview data were analysed before survey data (with the exception of parental delivery method preference to identify positive and negative cases for interview diversity) to allow inductive analysis, without survey results influencing the data analysis.

**TABLE 6:** Characteristics of participants interviewed.

Characteristic	*n*
Sex of parent	Female, 12; male, 1
Location	Metropolitan, 9; regional, 4
Parent of multiples	1
Parent‐reported developmental concerns	3
Infant age group	4 months, 2; 8 months, 8; 12 months, 3

#### Categories

Manifest content analysis identified four main categories that influenced parents' perceptions of telehealth use for the AIMS assessment. Each category is described below and supported by de‐identified quotes that capture strengths and challenges of telehealth and express the contrasting perspectives of parents. A separate category, named ‘strategies for success’, was also identified which provided clinicians with practical advice on how to implement the AIMS by telehealth, on the basis of parents' feedback. A visualization of the categories is shown in Figure 1.

**FIGURE 1:**
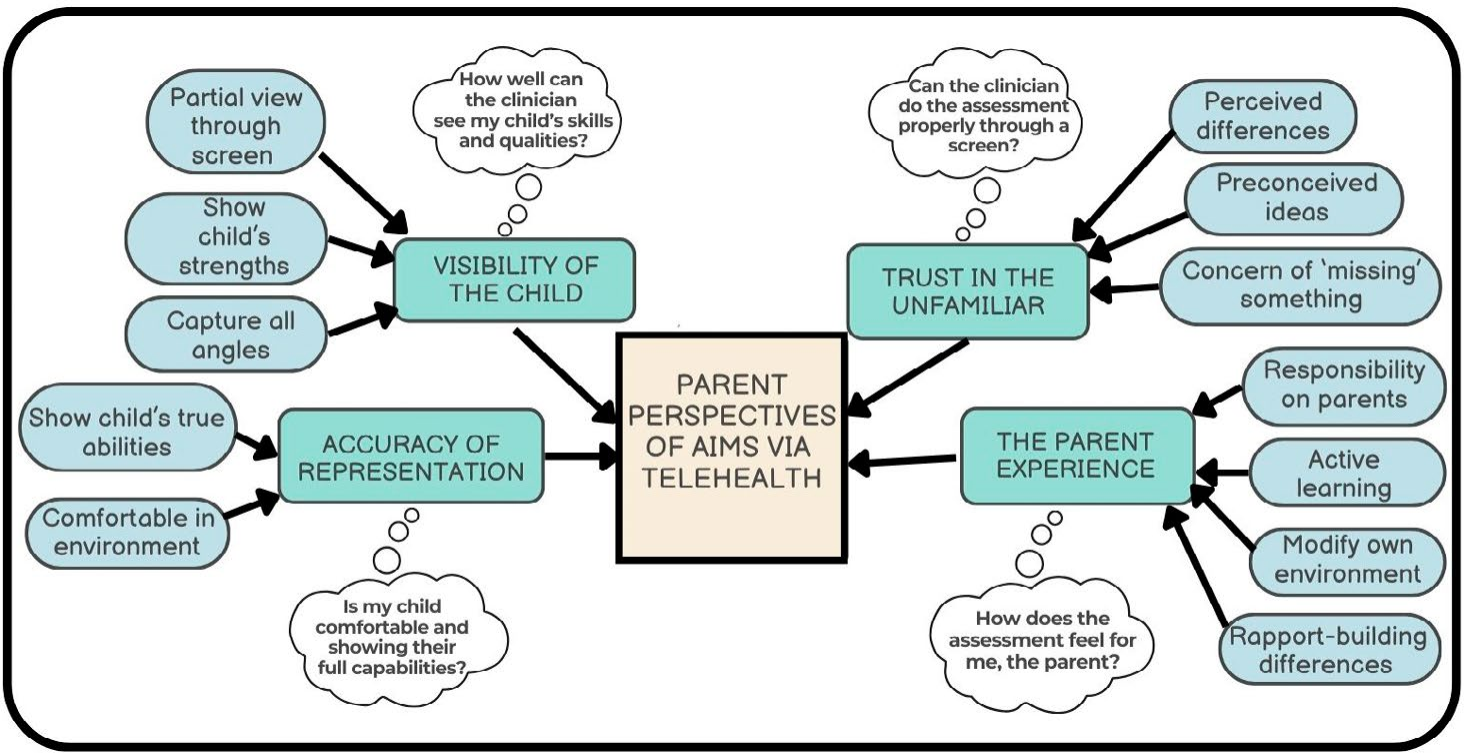
Visualization of category development.

##### Visibility of the child

This category questioned whether the clinician could see all of the child by telehealth: all their physical ability, personality, and other capabilities. Parents wanted the clinician to see how wonderful and unique their child was and to see strengths beyond their physical abilities. Parents were conscious that the telehealth assessor might only have had a partial view of their child through the screen and wanted the therapist to be able to see their child for who they were and all they were capable of doing, rather than what they could not do.

Because I knew that she had difficulties, it was really nice to see her interact with [the face‐to‐face clinician], and smile, and her engagement … probably has been one of her strengths. I don't know, it was just nice for [them] to see a strength of hers, and see her interact with [them] as opposed to just focusing on the stuff that she couldn't do, or had difficulty with. Parent 13 (resides regionally)

Some parents expressed insecurity about their ability to capture their child successfully ‘from all angles’ and talked about their desire to get ‘it right’. Overall, parents wanted to know, ‘Can you see? Like is it okay?’ (parent 11).

##### Accuracy of representation

This second category captured parents' desire for an accurate representation of their child's development. Parents wanted their child to feel comfortable and relaxed during the assessment so they played freely and were their natural selves, providing an accurate reflection of the child's abilities. For some children, the novelty factor of having a new face and new toys in the face‐to‐face assessment was motivational. For other children, the presence of an unfamiliar person inhibited their play and movement in the face‐to‐face session whereas the telehealth session allowed them to be in a familiar environment and showed the child ‘just … doing her thing’.

I'd lie her down and she'd just lie there. And like that's not what she does at all. And I think I felt a little bit stressed then because … like she's not doing anything, because she's just like there's someone else in my house. I don't know what to do. Which I think I was quite surprised by because I think I thought the telehealth would be harder… [In the face‐to‐face], she just either laid there or cried. And pretended that she has no movement in her arms or legs … not her normal behaviour at all … that's why I felt the telehealth was better, in that I felt like the physio saw a better representation of her … [she] felt much more comfortable not having someone in her space. Parent 12

##### Trust in the unfamiliar

There was a level of trust in a face‐to‐face assessment described by some parents that was not replicated by telehealth. Parents worried that something might be ‘missed’ by telehealth while one parent pointed out that ‘some clinicians might still not pick up on [the developmental concern], even in person’ (parent 3; resides regionally). Some parents referred to preconceived notions about physiotherapy being ‘hands on’ and that ‘face‐to‐face is better for movement’, while others approached the session with ‘curiosity around how is this going to work because I've always used [telehealth] for pure verbal communication’ (parent 7). Several parents expressed that if they had a particular developmental concern about their child, they would prefer a face‐to‐face assessment. However, all went on to say that they would still ‘believe’ the results of a telehealth assessment if the clinician raised developmental concerns.

I would assume the in‐person one is better quality but also that I would learn more from it … But if I had the telehealth one, I wouldn't be worried about it to the extent that I'd doubt the findings. Parent 10

##### The parental experience

This category described the experience of telehealth for the parents themselves. It encapsulated both the parents' perception of increased therapeutic responsibility in the assessment and the shift in the parent–therapist dynamic. Parents described a perceived increased responsibility to facilitate a successful interaction with their child during the telehealth assessment. For some parents, the active learning process they described through facilitating the telehealth assessment in their own environment, using their own resources, was helpful. They described gaining an understanding of their child's development, allowing them to better support their child's development through play. In contrast, other parents enjoyed watching the clinician interact with their child in person, learning from the way the clinician motivated or facilitated the child's movements during the assessment.

It was nice in‐person … to see the way the physio moved [my child] … they just did that quite comfortably and naturally and in a way that I hadn't been comfortable doing during the telehealth. Parent 5

Parents discussed the differences in the interaction and interpersonal connection felt between the clinician and parent during the telehealth and face‐to‐face assessments. While several parents felt the human connection and general ‘chit chat’ or ‘rapport building’ was reduced via the screen, this opinion was not unanimous. A few parents reported the telehealth session to be more relaxed with the clinician putting in more effort to build rapport via the screen while others did not note much difference between the two methods.

[Telehealth] definitely felt a bit more disconnected in the sense somebody is … there's less social interaction between me and the [clinician], and less back and forth … I don't know but it definitely felt a bit more just being observed which is a bit more uncomfortable … it does feel more engaged and engaging in person. That's a huge part of it … I remember the person who came to my house. I remember what she looked like … I remember her mannerisms, all that stuff. I can't remember a thing about the woman online. Parent 10

I really loved the telehealth appointment and I wonder if that's because the clinician had to work a little bit harder to establish rapport with me being on that telehealth interface … I think the in‐person session was … she was also wonderful but it was very perfunctory. She's coming to our house, she had somewhere to be, she probably had more assessments to do afterwards … that appointment was really professional, the second appointment was professional but also had that personal touch which I think made me really warm to the telehealth interface. Parent 7

Overall, even when telehealth was not the preferred delivery method, parents acknowledged the convenience and accessibility that telehealth offered.

It's incredible to think that [telehealth] assessment may be able to provide to families who are not only rural and remote families, but also just busy families … just as a new mum who is exhausted … you can just turn on the computer or on the phone and the assessment's underway. Parent 13 (resides regionally)

### Strategies for success

A separate category was formed collating feedback from parents about practical strategies for clinicians to optimize the telehealth experience (Table 7). Most parents raised the issue that they were unsure of what to ‘expect’ going into the telehealth session and gave suggestions about information that would be helpful to receive before the appointment. Responses were mixed about preferences for written or audiovisual information. Parents spoke of their experiences during the telehealth session, highlighting the importance of clear communication from the clinician to guide them through the assessment and explain ‘what’ and ‘why’. Parents liked frequent reassurance that they were doing it ‘right’ and liked to know when an assessment item had been completed (‘like “Oh good, yep”, “All right, we've got that one”—like more chatting would have reassured me that things were going okay I guess’ [parent 4]). The attributes of the clinician delivering the telehealth assessment were also influential in shaping parents' perceptions of telehealth (‘And I guess it's also clinician dependent, significantly a clinician‐dependent experience’ [parent 7]). Although most assessments occurred with just one parent present, the benefit of having a second adult was noted.

**TABLE 7:** Clinical strategies to assist successful implementation of the AIMS by telehealth.

**Before: establish expectations**	
Written information and/or short video provided previously	‘… like a short video that was sent to people, and be like, “These are types of things we'll be looking for,” so you sort of know what you're going to need to show. And something that might say, “Prepare some favourite toys,” or something like that … Yeah, just like [an infant] pulling themselves up, crawling, whatever, rolling over, that sort of stuff … everyone likes to know what to expect.’ Parent 2 (resides regionally)
Planning the environment on the basis of the child's level of mobility	‘… the telehealth one, because I didn't really know what things we would be doing, I didn't kind of know what room of the house to be in. Because she's only got one area that she was cruising along … It was just that it would have been a bit easier from my view if I knew what we were going to be doing beforehand.’ Parent 6 (resides regionally)
Benefit of face‐to‐face AIMS first	‘We loved the telehealth and maybe another factor would've been that it was our second time around so we sort of knew what we were expecting. I knew a little bit, I had a bit more confidence in what to do with [my child] and how to encourage [them] into different positions.’ Parent 7
**During: put parent at ease**	
Communication	‘Just at the beginning, like warning people that it might be a bit awkward. I'm not going to be constantly talking. If I'm silent, you know, just keep doing what you're doing. You're doing the right thing.’ Parent 4
Therapist influencing parents' experience	‘A general hello, this is who I am, this is what we're doing … but it was also delivered in such a way that it was just very comfortable and it certainly helped me to feel welcome in that space … The clinician was very professional and appropriate and explained everything and why. So I felt really confident in why I was being asked, or why [my child] was being asked to do certain things. Yes, generally, really positive experience.’ Parent 7
Benefit of two caregivers	‘My [partner] and I were both there and so it sort of did help that we were both there that we could sort of distract [our child] and listen to the physio … It might have been trickier if it was just one parent.’ Parent 8
**After: what's next?**	
Provide advice about the next steps	‘I think as long as I knew what my next steps were, to me, in person or telehealth wouldn't have made any difference. I was still able to ask questions and talk things through.’ Parent 2 (resides regionally)
Face‐to‐face follow‐up available if developmental concerns apparent	‘I think if the physio had said to me, I'm really worried about this, or I think there are some like developmental issues here, this is what I think you need to do, I think I would want to come in and … show me what to do in person … I think my confidence in supporting her wouldn't be high enough to have just the telehealth kind of explaining all the exercises, or all the things I should be doing.’ Parent 12
**Other considerations**	
Clinicians to assist parents in choosing face‐to‐face or telehealth assessment	‘If I was given the option, I feel like I would've picked in‐person … I don't think I would've really thought through that she might actually like [telehealth] better. So I almost feel like, you know, not a questionnaire but you almost put it to the parents, you know, what kind of temperament does your child have? What would you feel more comfortable with? What would they feel more comfortable with? Do they get overwhelmed in new spaces, or with new people? And do you think they might feel more comfortable if, the person wasn't in the room. And I guess giving them a bit of context about why telehealth might be a better fit. Because I still feel like telehealth in general, is still quite new in terms of people using it.’ Parent 12
Potential to use telehealth AIMS for waitlist screening/triaging	‘I think that would be perfect because I think then families get the sense that if everything is going okay then they get that result quicker, and if there are concerns, they know they're going to be fast tracked or prioritised. I can't see why a family wouldn't be interested in doing a telehealth for that reason if it's going to be more timely.’ Parent 5

Abbreviation: AIMS, Alberta Infant Motor Scale.

While the importance of choice for families between face‐to‐face and telehealth assessment was emphasized by many, several parents pointed out that decision‐making needed to be scaffolded by clinicians. Parents may need more information to fully understand the advantages and disadvantages of each delivery method and make the best choice for their child and family.

## DISCUSSION

This study explored parents' experiences of synchronous telehealth delivery of the AIMS in an Australian context. While most parents (89%) agreed or strongly agreed that telehealth is an acceptable way to complete the AIMS assessment, just over half (54%) preferred face‐to‐face assessment. Importantly, if a face‐to‐face assessment was not available, most parents (97%) would accept a telehealth appointment in the future.

Questionnaire and interview data highlighted the varying factors that influenced parents' perceptions of telehealth. Parents' experiences with the modalities were mixed, with some parents even surprising themselves with which delivery method they preferred. Parents residing in metropolitan areas responded slightly more favourably to telehealth than regional families, and those whose children had lower AIMS scores tended to prefer face‐to‐face assessment. Questionnaire responses commonly reported convenience, the child's comfort, and accessibility as benefits of telehealth, while the main challenges were camera positioning, technology issues, and parents' confidence in facilitating the assessment. Technology issues experienced were minor and did not prevent any assessments from occurring.

Interview data complemented questionnaire responses, further exploring and categorizing parents' experiences and the factors that shaped their perspectives of telehealth. Parents questioned how well the clinician could see their child's skills through the screen; how comfortable their child was to show their full capabilities; how much they trusted the modality; and how confident they felt to facilitate the assessment. Parents also shared practical strategies to assist clinicians with telehealth implementation, highlighting the importance of prior preparation, communication, and follow‐up.

Parents' opinions of delivery modality were varied. In clinical practice, assumptions therefore cannot be made about which method is more suitable for any given family. Although the study examined the delivery method rather than individual clinicians, it was evident that a parent's experience was influenced by the clinician's approach, particularly about the level of rapport and amount of reassurance provided.

Our findings are consistent with the reported acceptability of pre‐recorded (asynchronous) AIMS videos for parents in the Netherlands^10^ and synchronous telehealth for parents in Brazil.^4^ The use of synchronous telehealth can overcome some of the barriers reported by parents who pre‐recorded videos. Boonzaaijer et al.^10^ described the challenges that parents faced uploading the videos to a secure server and the time spent planning an appropriate moment to self‐record the videos. By conducting the assessment in real‐time, this alleviated any parental burden to upload videos. Scheduling the telehealth appointment, in the way that any health appointment would be prearranged, also removed the mental load of having to self‐select the perfect moment to record the assessment. Both the parents in this study and those who pre‐recorded videos^10^ experienced uncertainty and self‐doubt about capturing their child's movements as intended and wanting to do it ‘right’. Both groups also desired the child to be shown at their best, showing their full capabilities. The Brazilian synchronous telehealth study^4^ used survey data alone to establish caregiver satisfaction with the remote assessment and reported that caregivers felt very or somewhat comfortable with the telehealth AIMS. Similar to our findings, a minority of infants were distracted by the camera or were in an unsuitable mood for the telehealth assessment. Other environmental distractions (e.g. noise or other people) were raised as a challenge in the Brazilian study yet was not a significant concern for Australian parents.

Clinically, synchronous telehealth AIMS delivery shows promise in offering a successful alternative to face‐to‐face AIMS assessment, particularly when in‐person consultation is difficult or not possible, for example because of distance, illness, or service capacity. To assist implementation into clinical practice and optimize the experience for parents, findings from this study have been collated into a practical guide for clinicians (Figure 2). In addition, study findings highlighted the importance of offering families choice while providing clarity on the equivalence of the delivery methods and assisting parents in their decision‐making. The choice for telehealth extends beyond geographical location and should include consideration of the parents' preferences, their child's temperament, and other contextual factors such as family circumstances, preconceived perceptions of telehealth, and access and confidence with technology.

**FIGURE 2:**
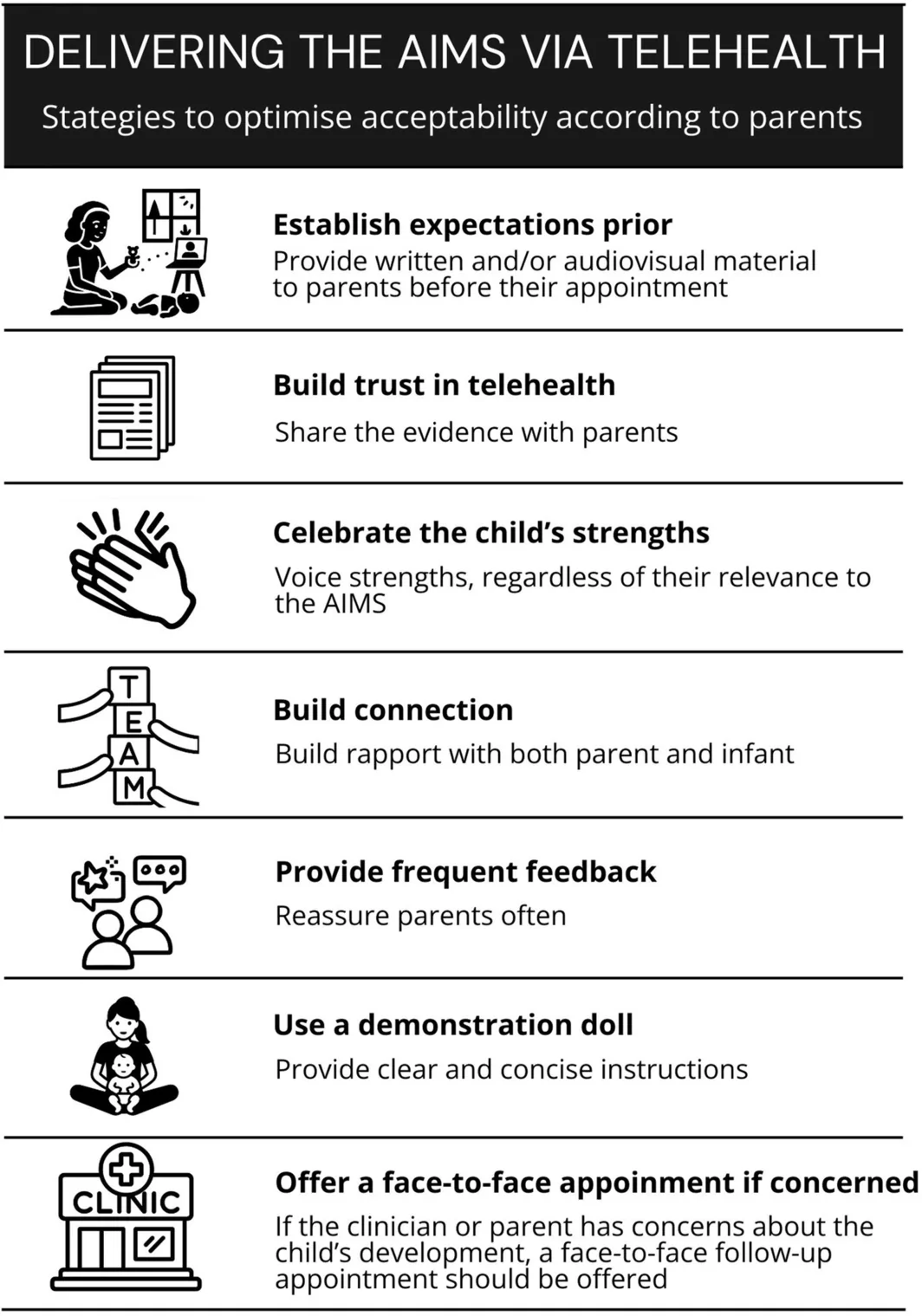
A practical guide for clinicians to implement the Alberta Infant Motor Scale (AIMS) into practice.

A unique feature of the study was the inclusion of participants from both metropolitan and regional locations. The mixed‐methods design was a study strength, allowing the inclusion of many parents' perspectives with a high survey response rate, accompanied by rich interview data for a more in‐depth understanding of the parent experience. This approach gave the opportunity to triangulate data findings to improve trustworthiness. The analysis approach involved strategies to ensure high‐quality analysis including reflexivity and the involvement of a second analyst.^20^, ^21^ To improve transferability of results, a maximum variation purposive sampling strategy was used to ensure diversity in the interview sample. As is the nature of qualitative research, we do not suggest the findings are representative of all families.^22^ Study participants were predominantly from formally educated backgrounds, had sufficient access to technology, and had expressed a willingness to participate.

A limitation of this study was that the interviewer (KLR) also completed many of the AIMS assessments. Several of the interview participants had previously met KLR as a clinician, therefore social desirability may have influenced the interview data. While response rates were excellent, selection and non‐response bias might also have limited the findings because of families being required to opt in to the study and 17 of the 117 parents not responding. It is also important to note that the face‐to‐face assessments took place in the home environment and not in a clinical setting. Responses in this study therefore represent the comparison between telehealth and home‐based assessments. This does not always reflect standard clinical practice and may have influenced parents' views on telehealth as they did not have to travel for the face‐to‐face assessment and the child was viewed in their home environment in both settings. Future research should compare parents' perceptions of clinic‐based versus telehealth assessments. Additionally, while not explored in this study, future research should consider an economic analysis of telehealth that includes costs both to parents and to health services. The timing of this study (which was shortly after the COVID‐19 pandemic) should be noted, as this may have influenced parents' perceptions of remote health care access (positively or negatively on the basis of individual experiences).

## CONCLUSION

Telehealth delivery of the AIMS is an acceptable method of infant gross motor assessment, according to Australian parents. However, around half of parents preferred face‐to‐face assessment. Preference of delivery method was variable and largely unpredictable. A slightly higher percentage of families living regionally and those whose children scored lower on the AIMS preferred face‐to‐face assessment. Parents generally liked the convenience and accessibility of telehealth but questioned whether the clinician could see all of their child through the screen, both visually and in regard to their capabilities. Clinical recommendations, based on parent feedback, have been provided to optimize parents' comfort and confidence using telehealth. The findings suggest that families be provided with choice between face‐to‐face and telehealth AIMS assessment and that decision‐making is supported by clinicians, taking into account family circumstances and considering that factors that shape the parents' telehealth experience. Future research should explore the use of telehealth with participants who reflect a greater diversity of socioeconomic, cultural, and educational backgrounds.

## FUNDING INFORMATION

This work was supported by an Australian Physiotherapy Association's Physiotherapy Research Foundation seeding grant and grants from the National Health and Medical Research Council of Australia (Centre of Clinical Research Excellence #546519; Centre of Research Excellence #1060733 and #1153176; Project Grant #108702; Leadership Level 1 #2016390 to JLYC), and the Victorian Government's Operational Infrastructure Support Program. KLR is supported by the University of Melbourne's Fay Marles Scholarship. None of the authors have any financial disclosures to declare.

## CONFLICT OF INTEREST STATEMENT

AJS is associate editor of the AIMS manual (*Motor Assessment of the Developing Infant*).

## Supporting information


**Appendix S1:** Caregiver survey.

**Appendix S2:** Semi‐structured interview guide.

## Data Availability

The data that support the findings of this study are available from the corresponding author upon reasonable request.

## References

[1] Piper M , Darrah J . Motor Assessment of the Developing Infant: Elsevier Health Sciences; 2021.

[2] Eliks M , Gajewska E . The Alberta Infant Motor Scale: A tool for the assessment of motor aspects of neurodevelopment in infancy and early childhood. Frontiers in neurology. 2022;13:927502. 10.3389/fneur.2022.927502 PMC9515325 36188401

[3] Spittle AJ , Doyle LW , Boyd RN . A systematic review of the clinimetric properties of neuromotor assessments for preterm infants during the first year of life. Developmental Medicine & Child Neurology. 2008;50(4):254–66. 10.1111/j.1469-8749.2008.02025.x 18190538

[4] Passamani RS , Shigihara CK , Gomes PG , dos Santos AN . Agreement of synchronous remote and in‐person application of the Alberta Infant Motor Scale: Cohort study. Journal of Telemedicine and Telecare. 2024;0(0):1357633X241245160. 10.1177/1357633X241245160 38659374

[5] Keane Y. Telepractice for early childhood intervention practitioners: Early Childhood Intervention Australia; 2020 [Available from: https://www.eciavic.org.au/resources/telehealth‐resources‐for‐eci‐providers.

[6] Curfman AL , Hackell JM , Herendeen NE , Alexander JJ , Marcin JP , Moskowitz WB , et al. Telehealth: Improving Access to and Quality of Pediatric Health Care. Pediatrics. 2021;148(3). 10.1542/peds.2021-053129 PMC9633975 34462339

[7] Cheung W , Aleman‐Tovar J , Johnston A , Little L , Burke M . A qualitative study exploring parental perceptions of telehealth in early intervention. Journal of Developmental and Physical Disabilities. 2023;35(3):353–73. 10.1007/s10882-022-09853-w PMC9244129 35789590

[8] Yang HW , Burke M , Isaacs S , Rios K , Schraml‐Block K , Aleman‐Tovar J , et al. Family perspectives toward using telehealth in early intervention. Journal of Developmental and Physical Disabilities. 2021;33:197–216.

[9] Wallisch A , Little L , Pope E , Dunn W . Parent Perspectives of an Occupational Therapy Telehealth Intervention. Int J Telerehabil. 2019;11(1):15–22. 10.5195/ijt.2019.6274 PMC6597151 31341543

[10] Boonzaaijer M , van Wesel F , Nuysink J , Volman MJM , Jongmans MJ . A home‐video method to assess infant gross motor development: parent perspectives on feasibility. BMC pediatrics. 2019;19(1):392‐. 10.1186/s12887-019-1779-x PMC6819354 31664955

[11] Gatlin RB , Hausch S , Caruso L . Commentary on “Concurrent Validity Between Live and Home Video Observations Using the Alberta Infant Motor Scale”. Pediatric physical therapy : the official publication of the Section on Pediatrics of the American Physical Therapy Association. 2017;29(2):152. 10.1097/PEP.0000000000000385 28350772

[12] Mendonça B , Kong M , Coombs A , Kysh L , Sargent B . Psychometric properties of the Alberta Infant Motor Scale and culturally adapted or translated versions when used for infant populations internationally: A systematic review. Dev Med Child Neurol. 2025;67(2):165–76. 10.1111/dmcn.16070 PMC11695772 39234875

[13] Kaushik V , Walsh CA . Pragmatism as a research paradigm and its implications for social work research. Social sciences. 2019;8(9):255.

[14] O'Brien BC , Harris IB , Beckman TJ , Reed DA , Cook DA . Standards for Reporting Qualitative Research: A Synthesis of Recommendations. Academic Medicine. 2014;89(9):1245–51. 10.1097/ACM.0000000000000388 24979285

[15] Rawnsley KL , Cheong JLY , Bennett K , Mahady ML , Smith SE , Zannino D , et al. Synchronous telehealth and face‐to‐face administration of the Alberta Infant Motor Scale. Dev Med Child Neurol. 2026;68(1):91–8. 10.1111/dmcn.16391 PMC12683301 40576266

[16] Etikan I. Comparison of Convenience Sampling and Purposive Sampling. American Journal of Theoretical and Applied Statistics. 2016;5:1.

[17] Elo S , Kyngäs H . The qualitative content analysis process. Journal of advanced nursing. 2008;62(1):107–15. 10.1111/j.1365-2648.2007.04569.x 18352969

[18] Elo S , Kääriäinen M , Kanste O , Pölkki T , Utriainen K , Kyngäs H . Qualitative Content Analysis:A Focus on Trustworthiness. Sage Open. 2014;4(1):2158244014522633.

[19] Rahimi S , khatooni M . Saturation in qualitative research: An evolutionary concept analysis. International Journal of Nursing Studies Advances. 2024;6:100174. 10.1016/j.ijnsa.2024.100174 PMC11080421 38746797

[20] Jack SM , Phoenix M . Qualitative health research in the fields of developmental medicine and child neurology. Developmental Medicine & Child Neurology. 2022;64(7):830–9. 10.1111/dmcn.15182 35156198

[21] Tracy SJ . Qualitative quality: Eight “big‐tent” criteria for excellent qualitative research. Qualitative inquiry. 2010;16(10):837–51.

[22] Willig C. EBOOK: introducing qualitative research in psychology: McGraw‐hill education (UK); 2013.

