# Supplementary material for: Acceptability of telehealth‐delivered Alberta Infant Motor Scale for Australian parents: A mixed‐methods analysis
Source: Dev Med Child Neurol. 2026 May 7;68(11):1610–20. doi: 10.1111/dmcn.70290 (PMC13649298; doi:10.1111/dmcn.70290)
Supplement: Supplementary file 1 [file DMCN-68-1610-s001.pdf]

Supplementary Material 2. Semi-structured Interview Guide

Theme	Guiding Questions	Prompts
**Introduction**<br><br>Setting the scene and building rapport	My brief background;<br>provide brief summary of the aims of the study; primary aim is to gather information regarding your experience with the AIMS assessment both in-person and via telehealth.<br><br>I appreciate you taking the time to talk to me today. We are really interested to hear your experiences. Anything that you think is relevant in this space would be fantastic to hear.<br><br>Permission to record (eg. So I don't miss anything that you tell me today, I will be recording the session. Are you happy to proceed?)<br><br>Our chat today is likely to take around one hour. Does that work for you or how much time do you have available?<br>If you need to stop or go at any point, just let me know.	
**Face to face Vs Telehealth experience**	What motivated you to become involved with our project? /Why did you choose to participate in this research?<br>(achieve their aims too from the interview).<br><br>Overall, what was your experience participating in the project like?<br><br>Tell me about your experience with the in-person physiotherapy assessment?<br><br>Tell me about your experience with the telehealth physiotherapy assessment?<br><br>Do you have a preference between in-person and telehealth delivery of the AIMS assessment? Why?<br><br>If you were to complete the assessment via telehealth again, is there anything that you would change or anything different that you would need for it to be successful?	What happened?<br>Tell me more about….<br>I'm interested to hear more…<br>How does that compare to your experience with…..
**Comfort with Telehealth**	Did you find any elements of the telehealth session particularly uncomfortable for you?<br><br>Was there anything that you would have preferred not to have to do?	Eg. pull to sit, supported sitting of baby, handling/positioning

	What would have made you feel more comfortable or confident about the telehealth session?<br><br>Assuming that you found aspects of the telehealth session helpful, can you describe what you found most beneficial about the telehealth assessment?<br><br>Tell me about the challenges of the telehealth session.	In the survey responses, we found that the most common benefit/challenge was…. Is that similar to your experience?
**Past experience/Future**<br><br><br><br>Preconceptions?	Have you ever received telehealth services outside of this research? Tell me about your experience.<br><br>Did you have any assumptions or thoughts about telehealth coming into this research? Did you anticipate that telehealth would be a good thing or a difficult experience?<br><br>If an in-person appointment wasn't an option (eg due to distance, illness, etc), how would you feel about being offered a telehealth appointment instead? What would you do in that circumstance?<br><br>Can you describe a situation where completing the assessment via telehealth wouldn't be suitable, in your opinion?<br><br>Do you have any advice for us so we can improve how the assessment is delivered via telehealth?	Would you attend? Would you request to wait for in person?
**Conclusion**	Is there anything further that you'd like to add?<br><br>Record/confirm demographics	

Please note: Semi-structured interviewing is an iterative process therefore the interview guide may be subject to minor modifications throughout the project
