# Supplementary material for: Acceptability of telehealth‐delivered Alberta Infant Motor Scale for Australian parents: A mixed‐methods analysis
Source: Dev Med Child Neurol. 2026 May 7;68(11):1610–20. doi: 10.1111/dmcn.70290 (PMC13649298; doi:10.1111/dmcn.70290)
Supplement: Supplementary file 2 [file DMCN-68-1610-s002.pdf]

Caregiver Survey: Agreement of face-to-face versus telehealth delivery of the Alberta Infant Motor Scale

Please complete the short survey below. We are interested to see if telehealth can be utilised for the AIMS assessment for clinical appointments in the future. Please share your thoughts and experience from participating in the AIMS study.

The survey should take less than 5 minutes to complete.

In your opinion, was telehealth an acceptable way to complete your child's assessment?

- ◯ Strongly agree
- ◯ Agree
- ◯ Neutral
- ◯ Disagree
- ◯ Strongly disagree

Please share your thoughts regarding why telehealth was not acceptable for you

____

Did you feel comfortable handling (ie. holding/moving/positioning) your child during the telehealth assessment?

- ◯ Strongly agree
- ◯ Agree
- ◯ Neutral
- ◯ Disagree
- ◯ Strongly disagree

What part/s of the assessment were uncomfortable for you? Why?

____

Did you feel confident that the physiotherapist was getting all the information they needed to complete the assessment via telehealth?

- ◯ Strongly agree
- ◯ Agree
- ◯ Neutral
- ◯ Disagree
- ◯ Strongly disagree

Please tell us why you weren't confident that the physiotherapist was getting everything they needed from the assessment via telehealth?

____

If given the option in future, would you choose an in-person or telehealth appointment for this assessment to be completed?

- ◯ In-person
- ◯ Telehealth
- ◯ No preference

Please share with us the reason for your choice

____

For future appointments, if an in-person appointment to complete the AIMS assessment wasn't possible, would a telehealth appointment be acceptable instead?

- ◯ Yes
- ◯ No

Please share with us why a telehealth appointment would not be acceptable?

____

In your opinion, what were the benefits (if any) of a telehealth AIMS assessment?

____

In your opinion, what were the challenges (if any) of a telehealth AIMS assessment?

____
